# Assessment of DNA damage by 53PB1 and pKu70 detection in peripheral blood lymphocytes by immunofluorescence and high-resolution transmission electron microscopy

**DOI:** 10.1007/s00066-020-01576-1

**Published:** 2020-01-31

**Authors:** Yvonne Lorat, Jochen Fleckenstein, Patric Görlinger, Christian Rübe, Claudia E. Rübe

**Affiliations:** 1 Department of Radiotherapy and Radiation Oncology, Medical Center, Saarland University, Homburg/Saar, Germany; 2 Department of Anesthesiology, DRK Hospitals Berlin Westend, Berlin, Germany

**Keywords:** Radiotherapy, Radio chemotherapy, DNA double-strand break, Phosphorylated Ku70, Chromatin

## Abstract

**Purpose:**

53BP1 foci detection in peripheral blood lymphocytes (PBLs) by immunofluorescence microscopy (IFM) is a sensitive and quantifiable DNA double-strand break (DSB) marker. In addition, high-resolution transmission electron microscopy (TEM) with immunogold labeling of 53BP1 and DSB-bound phosphorylated Ku70 (pKu70) can be used to determine the progression of the DNA repair process. To establish this TEM method in the PBLs of patients with cancer, we analyzed and characterized whether different modes of irradiation influence the formation of DSBs, and whether accompanying chemotherapy influences DSB formation.

**Methods:**

We obtained 86 blood samples before and 0.1, 0.5, and 24 h after irradiation from patients (*n* = 9) with head and neck or rectal cancers receiving radiotherapy (RT; *n* = 4) or radiochemotherapy (RCT; *n* = 5). 53BP1 foci were quantified by IFM. In addition, TEM was used to quantify gold-labelled pKu70 dimers and 53BP1 clusters within euchromatin and heterochromatin of PBLs.

**Results:**

IFM analyses showed that during radiation therapy, persistent 53BP1 foci in PBLs accumulated with increasing numbers of administered RT fractions. This 53BP1 foci accumulation was not influenced by the irradiation technique applied (3D conformal radiotherapy versus intensity-modulated radiotherapy), dose intensity per fraction, number of irradiation fields, or isodose volume. However, more 53BP1 foci were detected in PBLs of patients treated with accompanying chemotherapy. TEM analyses showed that DSBs, indicated by pKu70, were present for longer periods in PBLs of RCT patients than in PBLs of RT only patients. Moreover, not every residual 53BP1 focus was equivalent to a remaining DSB, since pKu70 was not present at every damage site. Persistent 53BP1 clusters, visualized by TEM, without colocalizing pKu70 likely indicate chromatin alterations after repair completion or, possibly, defective repair.

**Conclusion:**

IFM 53BP1 foci analyses alone are not adequate to determine individual repair capacity after irradiation of PBLs, as a DSB may be indicated by a 53BP1 focus but not every 53BP1 focus represents a DSB.

**Electronic supplementary material:**

The online version of this article (10.1007/s00066-020-01576-1) contains supplementary material, which is available to authorized users.

## Introduction

The effects of radiotherapy (RT) in cancer treatment can be significantly enhanced by simultaneous chemotherapy [1]. The action of both seems to depend on their ability to induce mutagenic and clastogenic DNA damage, including crosslinks, strand breaks, replication errors, and base adducts [2, 3], which can induce cell death. Precise dose distributions to the planning target volume (PTV) by highly conformal techniques are critical for minimizing side effects in adjacent organs at risk.

DNA damage repair mechanisms protect against adverse effects of carcinogenic therapies. While the likelihood of RT-induced side effects in organs at risk can be reliably assessed by dosimetric calculations, peripheral blood lymphocytes (PBL), especially in patients who received radiochemotherapy (RCT), are exposed to an erratic amount of events that may cause DNA damage. Double-strand break (DSB) repair is crucial for PBL survival following RT or RCT-induced DNA damaging effects. During nonhomologous end joining (NHEJ), the major mammalian DSB repair pathway, the Ku70–Ku80 heterodimer recognizes DSBs and maintains the broken DNA ends in close proximity until the DSB is rejoined [4]. In addition, the phosphorylated histone variant of H2AX, γH2AX, recruits repair proteins such as 53 binding protein 1 (53BP1) to the chromatin surrounding the DSB [5, 6]. 53BP1 is an important regulator in the cellular damage response to DSBs, promoting the binding of the distal DNA ends which occurs during variable diversity joining (V(D)J), class-switch recombination (CSR), or fusion of the unprotected telomeres. Recruitment of 53BP1 to the site of damaged chromatin also promotes nonhomologous end joining-mediated DSB repair (NHEJ) while preventing homologous recombination (HR) [7–10]. Specific primary and fluorescent secondary antibodies against 53BP1 localized to DSB repair foci [8, 11] may be used as markers to quantify DSB repair by immunofluorescence microscopy (IFM).

Assuming that each 53BP1 focus corresponds to one DSB, the number of foci in the nucleus can be applied to measure DNA damage caused by radiation exposure [12–18]. PBLs are suitable to assess the DNA damage response of patients, as peripheral blood samples can be taken repeatedly and at defined timepoints after irradiation. In addition, the hematopoietic system is radiosensitive and lymphocytes and their subpopulations are well characterized in terms of phenotype and function [19–22] and can be reliably isolated from blood [23]. Moreover, PBLs are in the resting state (G0) of the cell cycle [24, 25], thereby resulting in a prolonged presence of DNA damage [26–28].

Due to the limited resolution of IFM, 53BP1 visualization does not provide full-scale information regarding individual repair points and radiation sensitivity. Additionally, individual repair proteins of the Ku70–Ku80 heterodimer cannot be detected as their fluorescence is not sufficient to differentiate them from the background signal. The detection of both 53BP1 and the DSB-bound phosphorylated Ku70 (pKu70) would signal incomplete NHEJ repair sites. Here, high-resolution transmission electron microscopy (TEM) with gold-labeled pKu70 and 53BP1 [29, 30] was used to determine the suitability of this analysis for assessing individual PBL radiation sensitivity in patients with different tumor entities (head and neck or rectal cancers), isodose volumes, irradiation techniques (intensity modulated radiotherapy, IMRT or 3D-conformal RT, 3D-CRT), and treatment approaches (RT or RCT).

## Materials and methods

### Patients and treatment conditions

This study was conducted in accordance with the Helsinki declaration and with approval of the local ethics committee (Ärztekammer des Saarlandes). All patients (*n* = 9) signed written informed consent forms. Patients meeting the following inclusion criteria were enrolled between March 2011 and May 2012: Aged between 18 and 80 years; Karnofsky index >70%; completely resected head and neck squamous cell cancer (oral cavity, oropharynx, hypopharynx, or larynx) with postoperative RT indicated with or without chemotherapy; or diagnosis of rectal cancer with an indication for neoadjuvant or adjuvant pelvic radiotherapy (with or without chemotherapy). Patients with previous RT or chemotherapy and those with distant metastases were excluded.

All patients underwent standard computed tomography-based RT planning with 3D-conformal target volume delineation. IMRT with a predefined PTV arrangement of seven coplanar beam angles with 70 beam segments and standardized objectives based on the ICRU Report 83 [31] and constraints for normal tissues (brainstem, spinal cord, parotid glands, esophagus) based on QUANTEC data [32] with individualized clinical assessments was mandatory for patients with head and neck cancer (*n* = 4). A 60 Gy reference dose was prescribed to primary tumor sites and lymph node metastases in cervical regions and 50 Gy to non-involved cervical and supraclavicular lymph node regions. Single doses were 2.0 Gy, once daily, 5 days a week. Concomitant chemotherapy, if prescribed, included two cycles of cisplatin (20 mg/m^2^ intravenously over 0.5 h, D1–5; D29–33) and two cycles of 5‑fluorouracil (600 mg/m^2^ intravenously over 24 h, D1–5; D29–33). Patients with rectal cancer (*n* = 5) were treated in a prone position on a belly board by means of three 3D-conformal coplanar portals (0°, 90°, 270°) with a total reference dose of 50.4 Gy (optional 5.4 Gy boost to the primary tumor after 45 Gy) and a single dose of 1.8 Gy (once daily, 5 fractions/week). Concurrent neoadjuvant chemotherapy consisted of two cycles of 5‑fluorouracil (1000 mg/m^2^ intravenously over 24 h, D1–5; D29–33). Adjuvant 5‑fluorouracil was administered as a continuous infusion of 225 mg/m^2^ (D1–38). All patients were irradiated with a linear accelerator (ONCOR™ or ARTISTE™) from Siemens (Erlangen, Germany), using photons of 6 MV for IMRT of head and neck cancers or 18 MV for 3D-CRT of rectal cancers. The analysis of RT-related parameters included assessment of blood volume contained within the 50% isodose line (derived from volumetric computation of delineated blood vessels >1 cm in diameter), body volumes surrounded by the 10 Gy, 20 Gy, 30 Gy, and 45 Gy isodose lines (V10_iso_–V45_iso_), and coverage of the PTV (D80, D90).

### Blood sampling

For IFM analysis, blood samples were collected from a cubital vein in heparin-containing vials at 37 °C and diluted 1:2 with prewarmed Roswell Park Memorial Institute (RPMI) 1640 medium (Biochrom; Berlin, Germany) for immediate processing. All patient samples were obtained immediately before and 0.5 h after the first RT fraction (control and induction values, respectively) and 24 h after the first and fourth RT fractions in weeks 1, 2, 4, and 6 (after fractions 1, 4, 6, 9, 16, 19, and 26; and after fraction 29 in head and neck cancer samples).

To perform TEM analysis, blood samples were collected directly before and 0.1, 0.5, and 24 h after the first RT fraction for immediate processing.

For ex vivo experiments, blood from healthy donors was obtained, PBLs isolated, homogeneously irradiated, and incubated in RPMI at 37 °C.

#### Dose dependence

PBLs were suspended in cold phosphate-buffered saline (PBS), irradiated with different doses (0.5, 1.0, 2.0, or 4.0 Gy), and suspended in RMPI medium (Sigma-Aldrich, St. Louis, MO, USA) prior to a 0.5 h incubation at 37 °C allowing for repair.

#### Time course

Following irradiation with 1.0 Gy, PBLs were incubated in RPMI medium at 37 °C, and fixated 0.1, 0.25, 2.5, 8.0, and 24 h after irradiation. Nonirradiated PBLs from the same donor served as control.

### Blood sample preparation for IFM and TEM

Briefly, blood samples in heparin tubes were diluted with 6 ml RPMI and incubated at 37 °C. PBLs were isolated using a kit (PAA Laboratories; Cölbe, Germany). Blood samples were layered on Percol 400 and centrifuged at 1200 g for 20 min. 5 ml PBS was added to the resulting interphase and centrifuged at 300 g for 10 min. The separation yielded ~80% PBLs, ~15% monocytes, and ~5% granulocytes.

For IFM, PBLs were fixed in 100% methanol for 0.5 h and permeabilized in 100% acetone for 1 min at −20 °C. After washing cells in PBS with 1% fetal calf serum for 1 × 10 min at room temperature, samples were incubated with 53BP1 antibody (anti-53BP1, mouse monoclonal; Merck, Darmstadt, Germany) followed by a secondary fluorescent antibody (AlexaFluor-488, Invitrogen, Karlsruhe, Germany). Samples were mounted using Vectashield mounting medium (Vector Laboratories, Burlingame, CA, USA) with DAPI (4′,6-diamidino-2-phenylindole). Fluorescent images were captured and visually analyzed. A trained staff member identified and counted the cells until at least 300 cells and 40 foci for each timepoint were registered. All PBLs in each field of view were analyzed, even those without evidence of radiation damage.

For TEM, PBL pellets were fixed overnight with 2% paraformaldehyde and 0.05% glutaraldehyde in PBS. The ethanol-dehydrated samples were infiltrated with LR Gold resin (EMS, Hatfield, PA, USA). Afterwards, samples were embedded in resin containing 0.1% benzyl and kept for 24 h at −20 °C followed by ultraviolet light exposure until resin was polymerized. Ultrathin 70 nm slices were sectioned off the samples using a Microtome Ultracut UCT (Leica, Biel, Switzerland), picked up on pioloform-coated nickel grids, and processed for immunogold labeling. To block nonspecific staining, sections were floated on drops of 50 mM glycine and blocking solution. Afterwards, following rinsing, sections were incubated with different primary antibodies (53BP1 or pKu70 [anti-pKu70, rabbit polyclonal, pSer5; Abcam, Cambridge, UK]) overnight at 4 °C. The same primary antibodies used in fluorescence microscopy were applied in combination with gold-labeled secondary antibodies for TEM experiments in order to visualize pKu70 and detect incomplete DNA damage repair sites. A single IFM focus has a diameter of approximately 1.0 µm (Supplementary Figure 8a; green circle). When using gold-labeled secondary antibodies in the same IFM approach, this focus consists of two 53BP1 clusters, each one with a diameter of only 500 nm (Supplementary Figure 8b; red circles). In TEM analysis, this focal area can be subdivided further into euchromatic and heterochromatic compartments (Supplementary Figure 8c) and thus allows for reliable detection and quantification of DNA repair factors (Supplementary Figure 8d; pKu70, 10 nm, gold beads colored in red; 53BP1, 6 nm, colored in green) and their localization within different chromatin compartments.

After rinsing, goat secondary antibodies conjugated with 6 and 10 nm gold particles (EMS) were applied to the sections on the grids and then incubated for 1.5 h at room temperature. Subsequently, sections were washed and fixated with 2% glutaraldehyde in PBS. All sections were stained with uranyl acetate and examined with a Tecnai Biotwin^TM^ transmission electron microscope (FEI, Eindhoven, the Netherlands). For quantification, we identified pKu70 dimers (two 10 nm gold particles) and 53BP1 bead clusters (6 nm) visually at 48,000–86,000 × magnification and counted these in 50 randomly chosen nuclear sections.

### Statistical analysis

A one-sided Mann–Whitney test was performed using the statistical software OriginPro (version 8.5, OriginLab Corporation, Northampton, USA) to evaluate potential differences between data groups. The criterion for statistical significance was *p* ≤ 0.05.

The dispersion index test was used to determine the deviation of foci per cell distribution at the 0.5 h datapoint from Poisson statistics to demonstrate that—in the setting of partial body irradiation to the head and neck or pelvic region—only a proportion of PBLs was exposed to irradiation [33, 34]. The test was performed with the software Dose Estimate, version 3.0 (Chilton, UK).

## Results

To characterize the ongoing DNA repair process, PBLs from nine individuals with head and neck or rectal cancer (5 patients received RCT and 4 RT without chemotherapy) were analyzed by IFM and TEM. Table 1 shows the patients’ characteristics.

**Table 1:** Patients and treatment characteristics according to cancer type

Category	Head & neck cancer	Rectal cancer
*Total no. of patients*	4	5
*Age, years*		
Mean ± SD	64 ± 5	60 ± 10
Range	60–71	50–74
*Sex, no. (%)*		
Male	3 (75)	3 (60)
Female	1 (25)	2 (40)
*KPS, no. (%)*		
70	–	1 (20)
80	4 (100)	2 (40)
90	–	1 (20)
100	–	1 (20)
*T stage, no. (%)*		
T1	1 (25)	–
T2	3 (75)	–
T3	–	4 (80)
T4	–	1 (20)
*N stage, no. (%)*		
N0	2 (50)	2 (40)
N1	–	1 (20)
N2	2 (50)	2 (40)
*Chemotherapy*^*a*^*, no. (%)*	1 (25)	4 (80)
*Irradiation technique*	IMRT (6 MV photons)	3D-CRT (18 MV photons)
*Irradiation time per fraction*		
Mean	30 min	7 min
*Total dose, no (%)*		
46.8 Gy	–	1 (20)
50.4 Gy	–	4 (80)
60.0 Gy	4 (100)	–
*PTV, cm*^*3*^		
Mean ± SD	1047 ± 226	1411 ± 444
*D90-PTV, % ref. dose* *±* *SD*	89 ± 6	94 ± 5
*D80-PTV, % ref. dose* *±* *SD*	95 ± 2	97 ± 3
*Isodose volume, cm*^*3*^		
V5_iso_ ± SD	6703 ± 1876	11,961 ± 1411
V10_iso_ ± SD	5184 ± 1447	10,030 ± 1012
V20_iso_ ± SD	3872 ± 924	7099 ± 647
V30_iso_ ± SD	2704 ± 627	5575 ± 510
V45_iso_ ± SD	1427 ± 384	2183 ± 703
*Blood volume*^*b*^*, cm*^*3*^ *±* *SD*	117 ± 27	173 ± 44

*SD* standard deviation, *KPS* Karnofsky performance score, *PTV* planning target volume, *T* tumor, *N* node, *IMRT* intensity modulated radiotherapy, *3D-CRT* 3D-conformal radiotherapy, *D* dose

^a^Concurrent chemotherapy regime as described in “Materials and methods”, three patients with rectal cancer received a neoadjuvant regimen, one patient received an adjuvant regimen

^b^Derived from delineated blood vessels >1 cm in diameter and encompassed by the 50% isodose line

**Fig. 1:**
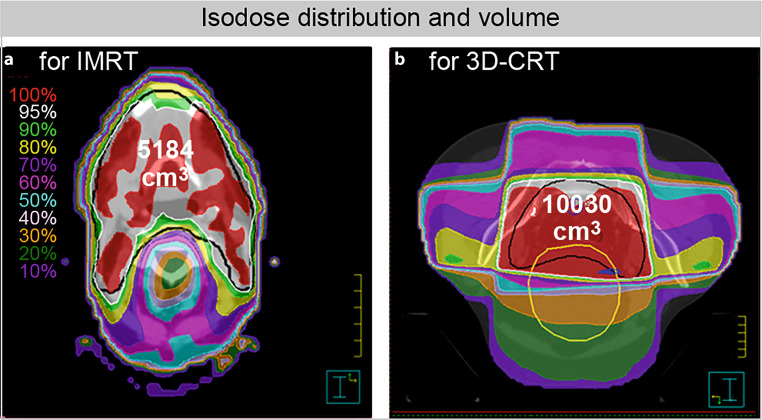
Representative isodose distribution. **a** IMRT was mandatory for patients with head and neck cancer, with a predefined arrangement of seven coplanar beam angles; **b** for patients with rectal cancer, through 3D-CRT with three coplanar portals. The irradiated volume within the *violet *isodose (10% reference dose) varies in size, depending on cancer type and irradiation modes. *IMRT* intensity modulated radiotherapy, *3D-CRT* 3D-conformal radiotherapy

Based on our assumption that the number of irradiation-induced DSBs depends on the applied dose and irradiation time, patients were grouped according to cancer type and the technique applied (patients with head and neck cancers received IMRT while those with rectal cancers underwent 3D-CRT; Fig. 1a, b). To show the influence of chemotherapy on DSB formation, patients were further divided into those who received chemotherapy (*n* = 5) and those who did not (*n* = 4). In total, 40 samples from patients with head and neck cancer and 45 from patients with rectal cancer (three technical replicates per sample) were analyzed.

Quantification of initial foci induction by IFM was completed on samples taken 0.5 h after the first RT fraction. 53BP1 foci were not detected in 39 of 432 PBLs (~10%) from patients with head and neck cancer and in 50 of 517 (~10%) from those with rectal cancer, confirming that partial-body irradiation causes limited PBL exposure (Table 2). In contrast, no 53BP1 foci could be detected in 85% of the unirradiated PBLs (in total 2735 from 3222 cells) taken before the first fraction.

**Table 2:** Dispersion analysis of 53BP1 foci distribution 0.5 h after the first RT fraction as measured by immunofluorescence

Tumor entity	53BP1 yield, foci per cell								
	0	1	2	3	4	5	6	7	≥8
**Head & neck cancer***, *no. of cells									
*Observed distribution*^a^									
Mean	39	60	61	91	55	52	26	21	27
±SD	1.38	1.45	1.83	2.83	2.31	1.76	1.62	1.46	1.64
*Poisson distribution*									
Mean	14	48	82	94	80	56	32	16	4
±SD	1.23	1.36	1.24	1.45	1.53	0.87	1.08	1.07	0.34
**Rectal cancer**, no. of cells									
*Observed distribution*^b^									
Mean	50	66	103	100	81	47	32	17	21
±SD	0.92	1.15	1.51	1.45	1.37	1.01	1.14	0.86	0.89
*Poisson distribution*									
Mean	22	68	108	114	92	58	32	14	8
±SD	0.82	1.19	1.35	1.21	0.89	0.81	0.69	0.86	0.51

^a^432 PBLs were analyzed in four patients. The resulting distribution after homogeneous ex vivo irradiation significantly deviates from a Poisson distribution, indicating a partial body irradiation (mean dispersion index is 1.8 ± 0.1 (standard error of the mean), U value (standard normal deviate)) is 8.3, and irradiated fraction of cells is 93% as calculated with the contaminated Poisson method

^b^517 PBLs were analyzed in five patients. The resulting distribution after homogeneous ex vivo irradiation significantly deviates from a Poisson distribution, indicating a partial body irradiation (mean dispersion index is 1.6 ± 0.1, U value is 6.3, and irradiated fraction of cells is 93% as calculated with the contaminated Poisson method)

To compare the appearance of 53BP1 foci among samples from different treatment types, we looked at the PTV size, radiation duration, and exposed blood volume from delineated blood vessels (>1 cm diameters) encompassed by the 50% isodose line. Table 2 shows the 53BP1 foci distribution analysis results measured by IFM 0.5 h after the first RT fraction (observed distribution).

This observed 53BP1 foci distribution did not correspond with the Poisson statistic as not all PBLs studied during in vivo radiation passed through the irradiation field and therefore foci were not detectable in all cells. To compare these results, PBLs from healthy donors (lab staff) were homogenously ex vivo irradiated with 2 Gy and 53BP1 foci quantified 0.5 h after irradiation. This 53BP1 foci distribution did match the Poisson statistic, as shown in Table 2 (Supplementary Table 3).

**Fig. 2:**
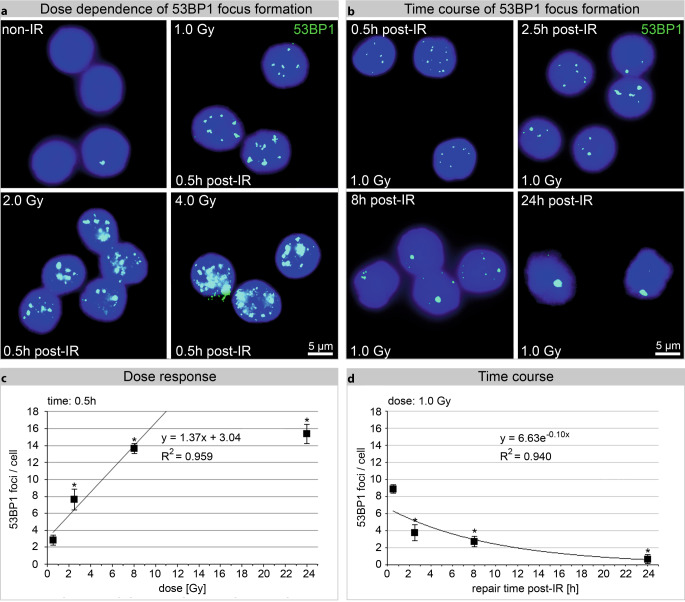
Dose dependence and time course of 53BP1 focus formation. **a** Immunofluorescence staining for 53BP1 in PBLs analyzed before (non-IR) and 0.5 h after homogeneous irradiation with 1.0, 2.0, or 4.0 Gy. **b** Time kinetics of radiation-induced 53BP1 foci. **c**, **d** 53BP1 was visually counted as number of foci/cells. All points are mean values of three different experiments where at least 300 cells were counted from 10 randomly chosen fields of view. *Asterisk*Statistically significant differences (*p* ≤ 0.05) compared with previous values. *R*^*2*^ coefficient of determination

In addition, a linear dose–response relationship up to 2.0 Gy was demonstrated 0.5 h after homogeneous irradiation (0.5–4.0 Gy), consistent with the literature [35, 36]. Representative images of cells fixed 0.5 h post irradiation with doses 0, 1, 2, and 4 Gy are shown in Fig. 2a and the associated quantitative dose response data in Fig. 2b. The time course of 53BP1 focus formation revealed a continuous 53BP1 foci loss until 24 h post exposure (1.0 Gy; Fig. 2c, d; Supplementary Table 4).

**Fig. 3:**
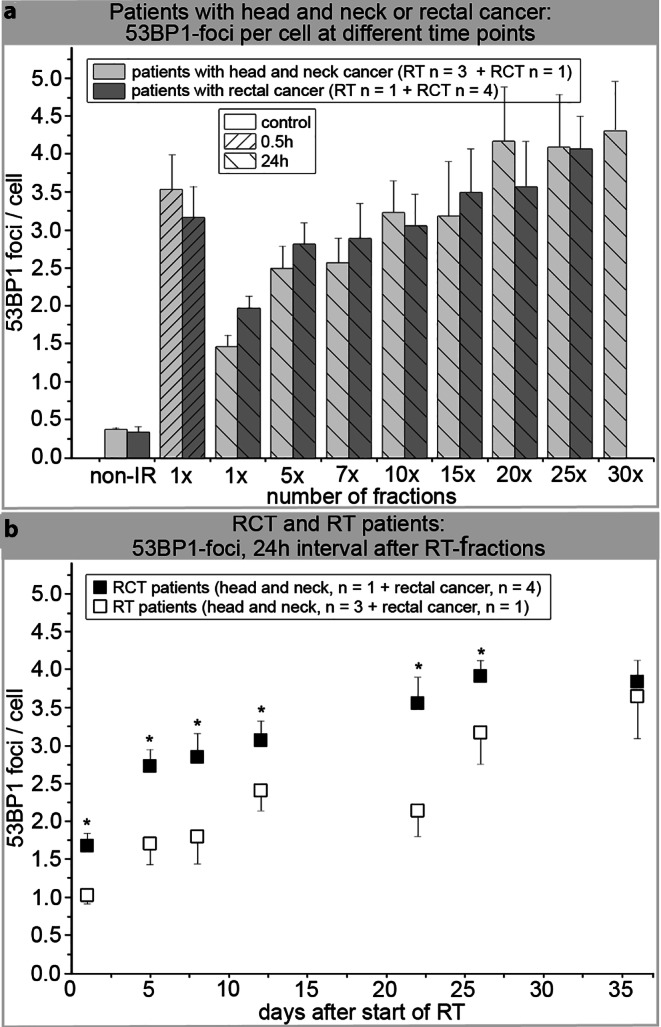
Quantification of 53BP1 foci by IFM. **a** The number of 53BP1 foci per PBL nucleus was counted before (non-IR) and 0.5 and 24 h after the first dose fraction (1 ×) as well as 24 h after a predefined number of additional in vivo irradiation fractions (5 × to 30 ×) to the head and neck (*n* = 4) or rectal (*n* = 5) regions. Data are presented as mean values of three technical replicates per patient ± standard error. **b** 24 h data during fractionated RT progression, stratified by the administration of concurrent chemotherapy. Data are presented as mean values of three technical replicates per patient ± standard error; the background number of foci/cell (control in week 1 before irradiation) was subtracted from the data. *Asterisk*Significant difference to RT patients (*p* ≤ 0.05)

Fig. 3a shows the number of 53BP1 foci per cell counted at each timepoint for all patients stratified by tumor entity using IFM.

The nonirradiated control (before RT) showed a low number of foci in patients with head and neck cancer (0.37 ± 0.02 53BP1 foci/cell) and in those with rectal cancer (0.33 ± 0.08 53BP1 foci/cell). At 0.5 h after the first RT fraction, we found a 10-fold rise in the 53BP1 foci number in head and neck cancer patient samples (3.54 ± 0.46 53BP1 foci/cell) and rectal cancer patient samples (3.17 ± 0.40 53BP1 foci/cell). Such a significant increase in the number of 53BP1 foci in PBLs of cancer patients after therapy commencement has also been described by Djuzenova et al. [37].

Over time, PBLs from both groups showed a decline in foci numbers, although it was still possible to visualize an average number of 1.46 ± 0.05 53BP1 foci/cell in PBLs from head and neck cancer patients and 1.97 ± 0.17 (~62%) 53BP1 foci/cell in PBLs from rectal cancer patients 24 h after the first fraction (1 ×; Fig. 3a).

With increasing numbers of administered RT fractions (up to 30 ×), the 53BP1 foci accumulated. Patients with head and neck cancer had 2.49 ± 0.30 53BP1 foci/cell (5 ×) 24 h after the first week of RT fractions and 4.31 ± 0.65 53BP1 foci/cell (30 ×) 24 h after further irradiation. Patients with rectal cancer had 2.81 ± 0.29 53BP1 foci/cell (5 ×) and 4.07 ± 0.44 53BP1 foci/cell (25 ×), respectively (Fig. 3a).

The 53BP1 foci levels in PBLs of patients with head and neck cancer tended to outnumber those of patients with rectal cancer; however, the differences were not significant. Moreover, we analyzed the number of 53BP1 foci according to whether patients received accompanying chemotherapy (Fig. 3b). Patients receiving chemotherapy had 1.69 ± 0.16 53BP1 foci/cell 24 h after the first RT fraction (1 ×) and those without chemotherapy had 1.02 ± 0.04 53BP1 foci/cell at the same timepoint. During the course of therapy, 53BP1 accumulated to 2.74 ± 0.08 foci/cell (5 days after start of RT) up to 3.78 ± 0.34 foci/cell (36 days after start of RT). Without accompanying chemotherapy, 53BP1 foci values were significantly lower, with 1.70 ± 0.24 53BP1 foci/cell (5 days after start of RT) up to 3.17 ± 0.86 53BP1-foci/cell (26 days after start of RT).

**Fig. 4:**
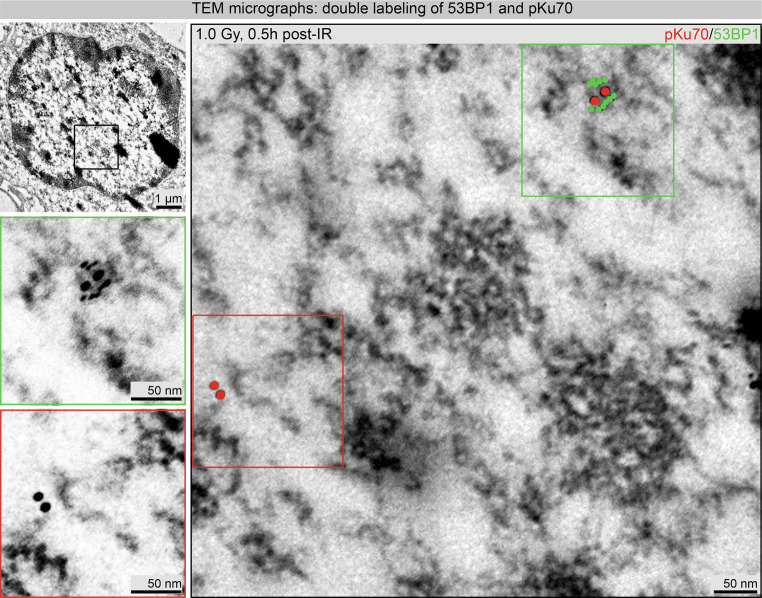
Visualization of pKu70 (10 nm beads, pseudo-colored in *red*) and 53BP1 (6 nm beads, pseudo-colored in *green*) 0.5 h after 1 Gy irradiation in a representative TEM image. Framed regions shown higher magnification in adjacent images

Quantification of 53BP1 foci by IFM enables estimation of DNA repair capacity after irradiation exposure. Application of TEM analysis improved the resolution of DNA damage patterns, which are obscured in IFM by the fluorescence of the labeled foci. Quantification of pKu70 and 53BP1 in PBLs after homogeneous ex vivo irradiation verified the suitability and reliability of the TEM method. Immunogold labeling of PBLs for pKu70 (10 nm bead size, colored in red) and 53BP1 (6 nm, colored in green) was completed 0.5 h after 1.0 Gy irradiation. Colocalization of 53BP1 clusters with pKu70 dimers was observed exclusively in heterochromatic areas. Additionally, pKu70 single beads and small 53BP1 clusters (2 to 5 beads) were occasionally present at the border between euchromatic and heterochromatic domains (Fig. 4).

**Fig. 5:**
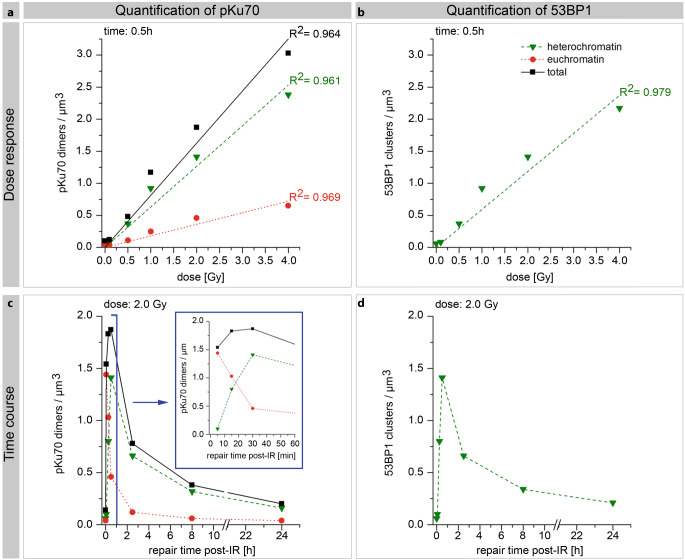
Quantification of pKu70 dimers and 53BP1 clusters by TEM after homogeneous irradiation with 1.0 Gy. Quantification of pKu70 dimers and 53BP1 clusters (quantified in ≥50 nuclear sections) in euchromatin (*red*) and heterochromatin (*green*) 0.5 h after irradiation with different doses (**a**, **b**) and at different timepoints after irradiation with 2 Gy (**c**, **d**). *R*^*2*^ coefficient of determination

In line with previous data [38], quantification of a dose response (0.1, 0.5, 1.0, 2.0, or 4.0 Gy) 0.5 h after irradiation revealed that the total number of pKu70 dimers and 53BP1 clusters was dose dependent (Fig. 5a, b). Gold bead dimers and clusters were normalized to nuclear area and section thickness (pKu70-dimers/µm^3^ or 53BP1 clusters/µm^3^).

A straight line correlation from 0.12 pKu70/µm^3^ (1.0 Gy) up to 3.03 pKu70/µm^3^ (4.0 Gy) with a background signal of 0.10 pKu70/µm^3^ in nonirradiated PBLs was demonstrated. The number of induced pKu70 dimers in euchromatin was consistently lower (from 0.04 pKu70/µm^3^ at 0.1 Gy to 0.65 pKu70/µm^3^ at 4.0 Gy) than those reached in heterochromatin (from 0.08 pKu70/µm^3^ to 2.38 pKu70/µm^3^). This indicates that 0.5 h after irradiation, portions of the originally induced euchromatic DNA damage were no longer present, whereas the highest recognition of heterochromatic DSBs took place at this timepoint. The numbers of 53BP1 clusters and pKu70 dimers almost correlate completely (from 0.08 53BP1/µm^3^ to 2.17 53BP1/µm^3^) due to their colocalization.

Analysis of the time kinetics occurred in the same manner, only at different time points (0.1, 0.25, 0.5, 2.5, 8.0, and 24 h) after homogeneous irradiation with 1.0 Gy. Highest values of pKu70 dimers were observed in euchromatic compartments after 0.1 h (1.44 pKu70/µm^3^). Ultimately, this value decreased to 1.03 pKu70/µm^3^ (~72%) after 0.25 h, to 0.46 pKu70/µm^3^ (~32%) after 0.5 h, to 0.12 pKu70/µm^3^ (~8%) after 2.5 h, to 0.06 pKu70/µm^3^ (~4%) after 5 h, and to 0.04 pKu70/µm^3^ (~3%) after 24 h (Fig. 5c). These results suggest that euchromatic DSBs are quickly recognized following irradiation and can be completely repaired within a few hours. On the contrary, the number of heterochromatic pKu70 dimers initially rose from 0.10 pKu70/µm^3^ (0.1 h post-IR) to 0.80 pKu70/µm^3^ (0.25 h post-IR) and to 1.41 pKu70/µm^3^ (0.5 h post-IR). Subsequently, the pKu70 dimers began to decrease in numbers 2.5 h after irradiation to 0.66 pKu70/µm^3^ (~47%), to 0.32 pKu70/µm^3^ (~23%) after 8 h, and to 0.16 pKu70/µm^3^ (~11%) after 24 h. The 53BP1 clusters showed roughly the same kinetics as the heterochromatic pKu70 dimers, with an increase from 0.10 53BP1/µm^3^ (0.1h post-RT) to 0.80 53BP1/µm^3^ (0.25h post-IR) and 1.41 53BP1/µm^3^ (0.5h post-IR). Then, 0.66 53BP1/µm^3^ (~47%) 53BP1 clusters were detectable after 2.5 h and decreased to 0.34 53BP1/µm^3^ (~24%) after 8 h, and 0.21 53BP1/µm^3^ (~15%) were still visible after 24 h (Fig. 5d). Additionally, 53BP1 clusters, consisting of 5 to 12 gold beads and without pKu70 colocalization, were observed 8 and 24 h after irradiation, potentially marking chromatin changes in areas where DNA damage was present.

**Fig. 6:**
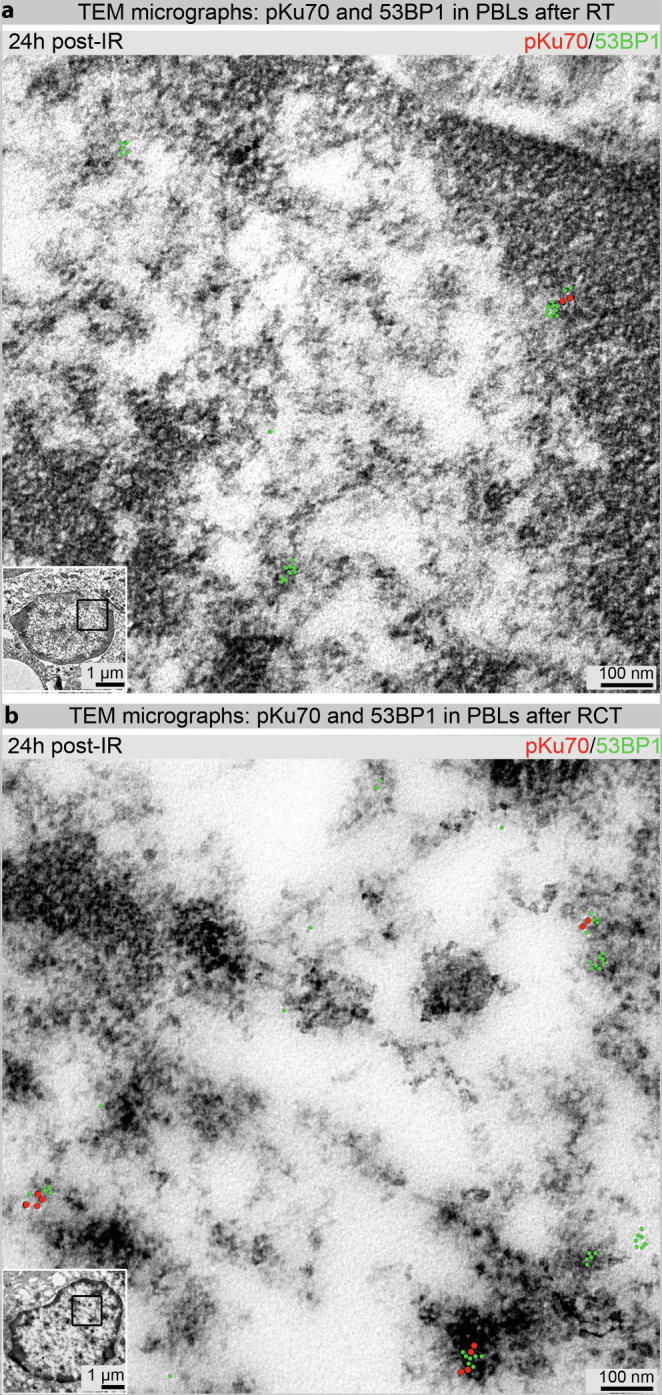
Visualization of pKu70 (10 nm beads, pseudo-colored in *red*) and 53BP1 (6 nm beads, pseudo-colored in *green*) in peripheral blood lymphocytes (*PBLs*) 24 h after first RT (**a**) and RCT (**b**)

To expand our knowledge on DNA damage, we investigated PBLs of patients with head and neck cancer after RT and RCT using TEM, before and 0.1, 0.5, and 24 h after the first fraction, by quantifying pKu70 dimers and 53BP1 clusters in euchromatin and heterochromatin of 50 nuclear sections per sample. Fig. 6a, b show representative TEM micrographs.

**Fig. 7:**
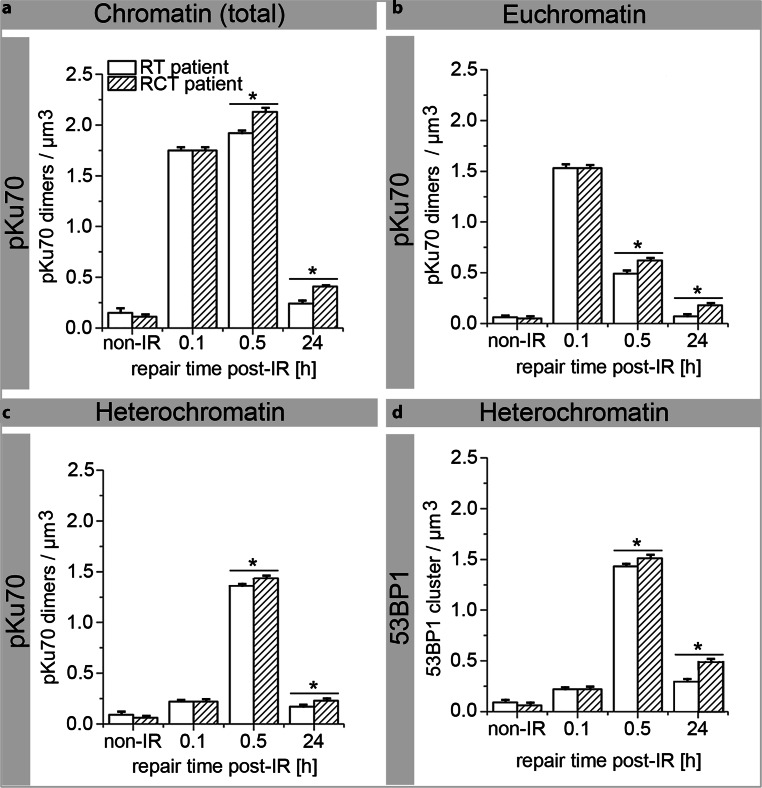
Quantification of pKu70 dimers and 53BP1 clusters by TEM in PBLs of patients who received RT or RCT. TEM quantification of pKu70 dimers per µm^3^ in total chromatin (**a**), euchromatin (**b**), and heterochromatin (**c**) following RT or RCT. Additionally, the number of 53BP1 beads per µm^3^ was quantified in PBLs of RT and RCT patients 0.1, 0.5, and 24 h after the first radiation fraction (**d**). *Asterisk* Statistically significant differences (*p* ≤ 0.05) compared with previous values

Visualization of 10 nm (pKu70 dimers) and 6 nm gold beads (53BP1 cluster) was improved by overlaying with red and green circles, respectively. The RCT patient showed a higher level of repair proteins than the RT patient in both chromatin domains (Fig. 7a–d) 0.5 and 24 h after the first fraction.

There was no difference between RT and RCT before and 0.1 h after in vivo irradiation in terms of the number of pKu70 dimers/μm^3^, with RT: 0.15 ± 0.01 and RCT: 0.11 ± 0.01 compared to RT: 1.75 ± 0.03 and RCT: 1.76 ± 0.04. However, detection of 53BP1 showed significantly higher values for RCT patients, both 0.5 and 24 h after in vivo irradiation (Fig. 7d and Supplementary Table 5).

## Discussion

In this study, we questioned whether the DNA damage repair in PBLs during radiotherapy for head and neck or rectal cancers is influenced by simultaneous chemotherapy or other variables, such as isodose volume, irradiation time, or by different irradiation techniques (IMRT or 3D-CRT). Additionally, we investigated how repetitive heterogeneous dose exposure influences the radiation-induced DNA damage of PBLs, especially in patients prescribed with concomitant chemotherapeutics. To do this, we quantified 53BP1 foci formation [13, 14] in irradiated PBLs, and found that not every 53BP1 focus equates to an unrepaired DSB.

IFM is a well-established method to visualize and analyze DNA repair proteins. It has the advantage of allowing fast examination of many cells. However, this is only possible for repair factors that accumulate in the vicinity of DSBs in sufficient number, thereby producing adequate fluorescent signals. IFM cannot be used to detect pKu70, which binds as a heterodimer with pKu80 at the ends of DSBs [4, 38]. Visualization of 53BP1 foci using IFM indicates DSBs; however, persisting 53BP1 foci mean fluorescence signals may still be detectable after the initial damage has been repaired. TEM enables clarification of the repair status of a DSB as it allows for detection of pKu70, the central repair protein of the NHEJ, which signals incomplete DNA damage repair.

Following homogeneous in vitro PBL irradiation, IFM showed a linear 53BP1 dose correlation 0.5 h post-IR up to 2 Gy (Fig. 2c) and TEM showed similar correlation up to 4 Gy (Fig. 5b). With increasing doses (>2 Gy), the fluorescent signal of individual adjacent foci overlapped, making IFM quantification difficult if not almost impossible (Fig. 2a). This limitation does not occur with quantitative TEM as gold beads were either present and quantifiable or absent. However, when LR Gold resin-embedded PBL sections are investigated and quantified using TEM, it is critical to always consider that only planar sections of the nucleus are examined and not the entire cell nucleus. To gain greater insight into the number of gold-labeled repair proteins in the entire nucleus, the labeling in 50-nuclei sections per dose or repair timepoint was quantified. Additionally, nonhomogeneously distributed pKu70 dimers and 53BP1 clusters in the cell nucleus over the number of nuclei sections was captured as reliably as possible by counting all 10 nm (pKu70) and 6 nm (53BP1) gold beads. The distance of a 10 nm gold bead from the antigen or repair protein is a maximum of 28 nm (Supplementary Figure 9). In contrast, IFM enabled visualization and quantification of foci in each entire PBL nucleus. The primary antibodies and the fluorochrome-coupled secondary antibodies penetrated the fixed cells and cell nuclei due to the permeabilization step (acetone, 1 min at −20 °C). In IFM, the degree to which cell structures were preserved after this chemical treatment was not detectable by means of the DAPI signal. No other publications have reported on the structural preservation and quality of cells after IFM sample preparation. After sectioning embedded PBLs, immunogold-labeled repair proteins were visualized and quantified in TEM. Permeabilization was not necessary, as antigens were found freely accessible near the surface. All cell structures (membranes, mitochondria, etc.) were perfectly visible in TEM and optimally preserved.

PBLs, which are often used for biological dosimetry [39] and for determination of individual radiosensitivity [40, 41], do not go through the cell cycle but remain in the G0 phase. Several studies have shown that PBLs sometimes undergo apoptosis 12–24 h after irradiation, which is characterized by significant chromatin condensation [35, 42]. This apoptotic chromatin condensation within human PBLs may prevent decomposition of residual DNA repair foci, which were observed in PBLs 24 h after irradiation [43]. Irradiation-induced residual foci in condensed chromatin can persist longer than 24 h in apoptotic G0 PBLs. Persisting foci are not to be confused with existing DSBs, as these especially slow repaired or unrepaired DSBs are eventually responsible for induction of apoptosis. Moreover, pulsed-field gel electrophoresis and confocal laser microscopy experiments have shown that in normal human fibroblasts, repair of existing DSBs does not correspond with the counted 53BP1 foci [44]. Our results confirm this, as 24 h after irradiation, 53BP1 clusters often did not colocalize with pKu70 (Fig. 6a, b). As we have already reported, DSBs can be visualized by pKu70 dimers [30, 38] that bind directly to the ends of DSBs. In TEM, lack of pKu70 within a 53BP1 cluster indicates the absence of a DSB at this point. Therefore, the frequency of colocalizations between pKu70 and 53BP1 is largely dependent on the point in time post irradiation. The recorded accumulation of 53BP1 foci by IFM in Fig. 3a, after an increasing number of applied fractions, is probably due to a mixture of newly induced DSBs per daily fraction and the generation of persistent chromatin changes after unfinished or defective DSB repairs. Loss of 53BP1 foci occurs through apoptosis of aged or damaged PBLs and the successful repair of DSBs [41, 44]. However, as an overall (but not linear) increase in the number of 53BP1 foci was observed, especially 24 h post-IR between the first (1 ×) and last (25 × or 30 ×) fraction, it is reasonable to assume that at this timepoint, accumulation of persisting 53BP1 foci was significantly involved, whereas during the previous 24 h, more repairable DSBs were eliminated. A continuous but not smooth increase in 53BP1 foci was detected throughout the entire treatment period—possibly due to a mixing of the opposing events of DSB induction, their repair, and the appearance of residual 53BP1 foci within a period of 24 h. Additionally, new PBLs formed over the period of 30 fractions. At the time of blood sampling, these may have crossed the irradiation field only once or even not at all, which is why, accordingly, no or only initial 53BP1 foci were detected in these PBLs. By contrast, initial and residual 53BP1 foci can be detected in older PBLs and moreover, older and damaged PBLs are preferentially eliminated through apoptosis. Furthermore, RT has immunomodulatory properties, the extent of which is determined by the radiation dose administered, the concomitant chemotherapy, and the immune system of the patient. As such, occurrence and repair of DNA damage in the PBLs of patients is determined by a range of influencing factors that are in turn influenced by external parameters (different techniques for application of radiation, isodose volumes, dose applied, duration of radiation application, and concomitant chemotherapy) and internal influencing factors (immune system, individual capacity for DNA repair) [45, 46]. Thus, it is not possible to evaluate the repair capacity based on a rise in 53BP1 foci detected 24 h after irradiation. Most persistent 53BP1 foci responsible for the increase are located exclusively in the periphery of heterochromatin domains and contain no pKu70. Most likely, these represent apoptotic processes rather than unrepaired DSBs. In Fig. 3b, we show higher 53BP1 foci levels for all patients who received RCT, independent of the collective. The cytotoxic effects of chemotherapy drugs have been described at length in the literature, whereby their objective is to induce DNA damage and activate apoptosis [47, 48].

By using the higher resolution of TEM in combination with the immunogold labeling of pKu70 and 53BP1 within the intact nuclear cell ultrastructure, we were able to detect a higher number of pKu70 dimers in the euchromatin and heterochromatin of PBLs in RCT patients than in those of patients after a single RT 24 h post-IR. In addition, we visualized pKu70 dimers individually as well as collectively (2 × or 3 × pKu70 dimers), which suggests multiple DSBs in close proximity (Fig. 6). These results indicate that the scale of DNA damage induced during radiotherapy is affected by the presence of accompanying chemotherapy. Thus, the number of DSBs in PBLs was not significantly influenced by the irradiation technique (IMRT or 3D-CRT) or the size of the irradiation field. This, however, could be due to the possibility that effects induced by IMRT, in which a smaller PTV is irradiated over a longer period of time, counterbalance those induced in 3D-CRT, in which a larger volume is irradiated over a shorter time period.

Based on these data, we propose that persisting DSBs (pKu70 dimers) represent more severe damage induced by RCT (1, 2, or more pKu70 dimers representing multiple DNA lesions). Repair seems to be difficult or even impossible in these cells. A large-scale study is necessary to be able to better study the cause of this observation, in particular to clarify how the concomitant chemotherapy prolongs the dwell time of existing DSBs in PBLs that are located in the G0 phase of the cell cycle. In addition, as persistent DSBs were detectable only at certain times >0.5 h after irradiation and always at the edge of heterochromatic domains, we suspect that cellular processes, such as the opening of densely packed heterochromatic regions containing one or more DSBs, delay repair. However, not every residual focus is equivalent to a remaining DSB, since pKu70 was not present at every damage site. Persistent 53BP1 clusters without colocalizing pKu70 are likely to show chromatin alterations after completion or possibly defective repair. Therefore, IFM 53BP1 foci analyses alone are not adequate to determine the individual repair capacity after the irradiation of PBLs, as a DSB may be marked by a 53BP1 focus but not every 53BP1 focus represents a DSB.

## Caption Electronic Supplementary Material

**Fig. 8** Different resolution powers of light and electron microscopy.** a** Immunofluorescent image of 53BP1 foci 0.5 h after irradiation with 1 Gy in the DAPI-stained nucleus of a peripheral blood lymphocyte. pKu70 cannot be observed by IFM. **b** Light microscopy image of a PBL nucleus. By virtue of antibodies targeting 53BP1, two clusters can be seen (red circles). **c** Electron microscopy image (TEM, 2700 × magnification). Euchromatin (bright) and heterochromatin (dark) can be clearly differentiated within the nucleus. **d** Reliable visualization of immunogold-labeled 53BP1 (green) and pKu70 (red) by means of TEM (48,000 ×)

**Fig. 9** 3D model (using computer-aided design, AutoCAD 2017, Autodesk GmbH, USA) of a primary antibody, bound to a secondary antibody coupled to a 10 nm colloidal gold particle

**Table 3** Dispersion analysis of 53BP1 foci distribution 0.5 h after the first RT fraction as measured by immunofluorescence. The resulting distribution after homogeneous ex vivo irradiation (2 Gy; 0.5 h) significantly deviates from a Poisson distribution, indicating a partial body irradiation

**Table 4** Induction: Quantification of 53BP1 foci per nucleus 0.5 h after irradiation (0.5, 1, 2, and 4 Gy). Repair: The number of 53BP1 foci per nucleus was enumerated at 0.5, 2.5, 8.0, and 24 h after 1 Gy

**Table 5** PBLs of patients with head and neck cancer after RT and RCT were investigated using TEM before and 0.1, 0.5, and 24 h after the first fraction by quantifying pKu70 dimers and 53BP1 clusters in euchromatin and heterochromatin of 50 randomly chosen nuclear sections. Gold bead dimers and clusters were normalized to nuclear area and section thickness (pKu70 dimers/µm^3^ or 53BP1 clusters/µm^3^) and presented as mean values
