# Supplementary figures and images for: Assessment of DNA damage by 53PB1 and pKu70 detection in peripheral blood lymphocytes by immunofluorescence and high-resolution transmission electron microscopy
Source: Strahlenther Onkol. 2020 Jan 31;196(9):821–33. doi: 10.1007/s00066-020-01576-1 (PMC7449954; doi:10.1007/s00066-020-01576-1)

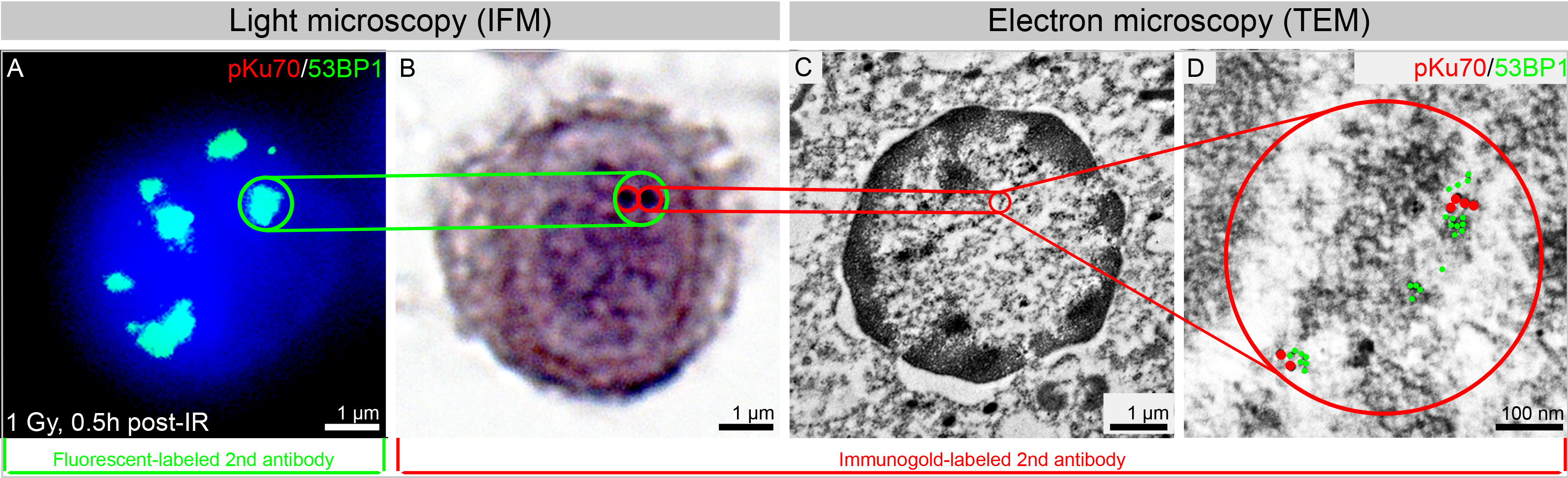

Supplement: Supplementary file 1 — Fig. 8 Different resolution powers of light and electron microscopy. a Immunofluorescent image of 53BP1 foci 0.5 h after irradiation with 1 Gy in the DAPI-stained nucleus of a peripheral blood lymphocyte. pKu70 cannot be observed by IFM. b Light microscopy image of a PBL nucleus. By virtue of antibodies targeting 53BP1, two clusters can be seen (red circles). c Electron microscopy image (TEM, 2700 × magnification). Euchromatin (bright) and heterochromatin (dark) can be clearly differentiated within the nucleus. d Reliable visualization of immunogold-labeled 53BP1 (green) and pKu70 (red) by means of TEM (48,000 ×) [file 66_2020_1576_MOESM1_ESM.tif]

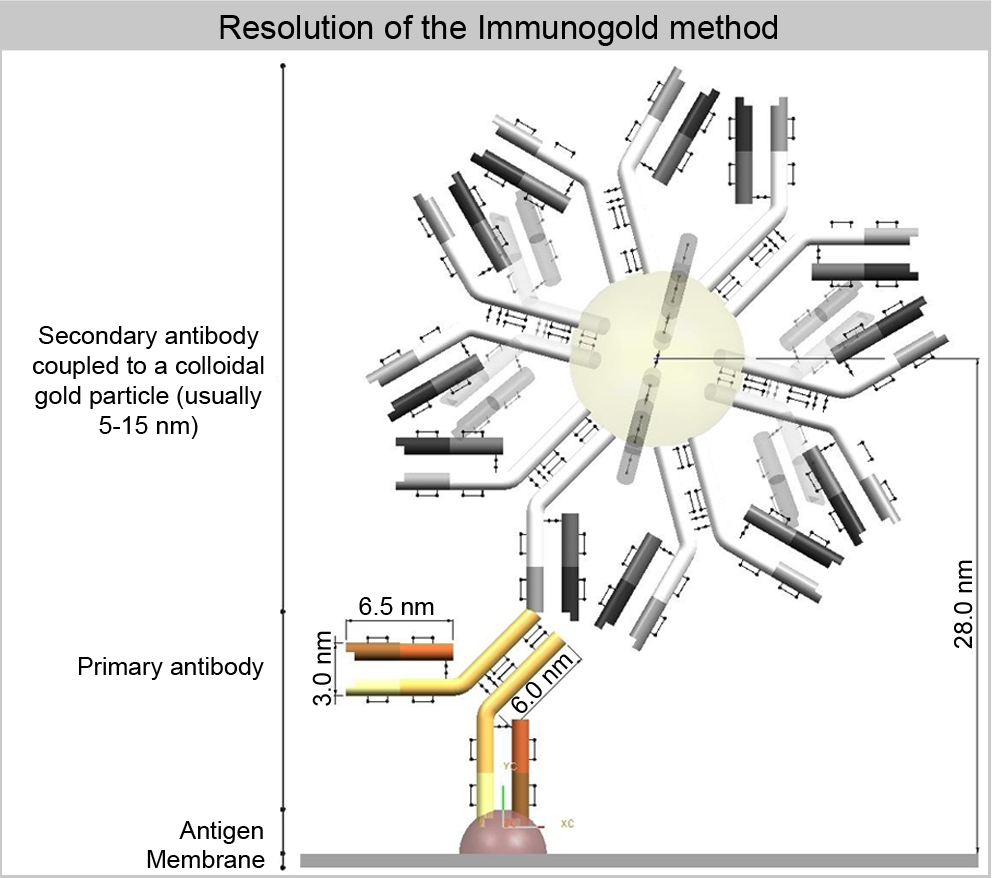

Supplement: Supplementary file 2 — Fig. 9 3D model (using computer-aided design, AutoCAD 2017, Autodesk GmbH, USA) of a primary antibody, bound to a secondary antibody coupled to a 10 nm colloidal gold particle [file 66_2020_1576_MOESM2_ESM.tif]
