# Supplementary material for: Assessment of DNA damage by 53PB1 and pKu70 detection in peripheral blood lymphocytes by immunofluorescence and high-resolution transmission electron microscopy
Source: Strahlenther Onkol. 2020 Jan 31;196(9):821–33. doi: 10.1007/s00066-020-01576-1 (PMC7449954; doi:10.1007/s00066-020-01576-1)
Supplement: Supplementary file 3 — Table 3 Dispersion analysis of 53BP1 foci distribution 0.5 h after the first RT fraction as measured by immunofluorescence. The resulting distribution after homogeneous ex vivo irradiation (2 Gy; 0.5 h) significantly deviates from a Poisson distribution, indicating a partial body irradiation [file 66_2020_1576_MOESM3_ESM.pdf]

Tumor entity	53BP1-yield, foci per cell								
	**0**	**1**	**2**	**3**	**4**	**5**	**6**	**7**	**≥8**
**Head&neck cancer**<br>no. of cells									
**Observed distribution**									
Mean	39	60	61	91	55	52	26	21	27
± SD	1.38	1.45	1.83	2.83	2.31	1.76	1.62	1.46	1.64
Patient 1	38	55	65	99	49	45	21	14	26
in-vivo	31	59	61	97	55	50	27	20	24
	42	60	59	107	59	49	32	12	19
Patient 2	44	67	67	90	57	53	25	24	29
in-vivo	48	59	69	99	66	59	22	20	35
	39	63	60	87	61	63	30	17	38
Patient 3	33	51	51	103	55	60	35	22	24
in-vivo	35	60	68	96	67	52	30	25	18
	41	68	70	84	62	56	28	18	30
Patient 4	44	52	52	81	43	49	17	26	24
in-vivo	41	63	54	79	48	47	16	29	31
	36	61	60	75	42	42	24	27	28
**Poisson distribution**									
Mean	14	48	82	94	80	56	32	16	4
± SD	1.23	1.36	1.24	1.45	1.53	0.87	1.08	1.07	0.34
Donor 1	21	56	56	99	75	59	28	10	4
ex-vivo	14	50	50	92	71	61	33	13	3
	16	51	51	98	78	53	30	14	5
Donor 2	9	43	43	86	85	56	35	20	4
ex-vivo	11	38	38	89	90	55	36	21	2
	15	46	46	92	83	51	39	17	3
Donor 3	10	42	42	94	77	58	32	11	5
ex-vivo	12	49	49	90	80	57	29	15	5
	8	50	50	91	72	59	34	18	2
Donor 4	18	49	49	105	84	52	28	16	5
ex-vivo	22	46	46	93	81	55	26	22	2
	15	52	52	98	82	53	29	19	3
**Rectal cancer**<br>no. of cells									
**Observed distribution**									
Mean	50	66	103	100	81	47	32	17	21
± SD	0.92	1.15	1.51	1.45	1.37	1.01	1.14	0.86	0.89
Patient 1	47	60	114	89	77	53	35	19	13
in-vivo	49	65	113	99	75	50	36	15	19
	51	67	109	93	84	47	30	14	25
Patient 2	53	70	105	103	80	41	32	23	17
in-vivo	58	69	97	100	82	49	25	20	27
	55	62	101	99	88	50	26	19	20
Patient 3	50	58	102	110	75	45	29	11	21
in-vivo	49	61	96	105	71	44	31	13	24
	53	65	99	100	79	40	26	16	17
Patient 4	50	60	100	98	80	49	34	17	15
in-vivo	51	67	104	101	86	50	32	21	20
	51	68	94	95	89	47	36	22	19

Patient 5	47	70	102	104	90	41	37	15	24
in-vivo	42	74	110	111	81	42	40	16	22
	50	69	105	99	79	49	37	19	26
**Poisson distribution**									
Mean	22	68	108	114	92	58	32	14	8
± SD	0.82	1.19	1.35	1.21	0.89	0.81	0.69	0.86	0.51
Donor 1	19	77	111	115	90	60	32	15	9
ex-vivo	20	72	112	118	94	55	29	10	8
	25	70	107	114	92	57	30	9	5
Donor 2	23	68	113	110	89	58	27	16	8
ex-vivo	19	62	100	116	92	62	33	19	7
	15	61	109	117	95	61	35	14	5
Donor 3	22	69	108	119	93	60	29	21	8
ex-vivo	24	73	99	107	97	55	33	18	4
	20	74	114	108	90	54	30	16	10
Donor 4	17	66	101	107	91	60	34	15	11
ex-vivo	25	63	110	110	99	62	36	16	8
	26	62	103	113	98	64	30	12	9
Donor 5	25	69	112	119	87	58	28	12	7
ex-vivo	20	66	115	120	89	53	35	10	6
	23	67	102	121	90	57	31	13	10
