# Supplementary material for: Assessment of DNA damage by 53PB1 and pKu70 detection in peripheral blood lymphocytes by immunofluorescence and high-resolution transmission electron microscopy
Source: Strahlenther Onkol. 2020 Jan 31;196(9):821–33. doi: 10.1007/s00066-020-01576-1 (PMC7449954; doi:10.1007/s00066-020-01576-1)
Supplement: Supplementary file 4 — Table 4 Induction: Quantification of 53BP1 foci per nucleus 0.5 h after irradiation (0.5, 1, 2, and 4 Gy). Repair: The number of 53BP1 foci per nucleus was enumerated at 0.5, 2.5, 8.0, and 24 h after 1 Gy [file 66_2020_1576_MOESM4_ESM.pdf]

**3C**	**Induction** [53BP1-foci/cell]
**Dose [Gy]**	
0.5	2.82 ± 0.016
1.0	7.69 ± 0.772
2.0	13.66 ± 0.155
4.0	15.60 ± 0.709

The unirradiated control (0.67±0.080) was subtracted

**3D**	**Repair** [53BP1-foci/cell]
**Time point after RT [h]**	
0.5	8.88 ± 0.456
2.5	3.77 ± 0.201
8.0	2.69 ± 0.123
24.0	0.61 ± 0.038

The unirradiated control (0.61 ± 0.014) was subtracted
