# Supplementary material for: Assessment of DNA damage by 53PB1 and pKu70 detection in peripheral blood lymphocytes by immunofluorescence and high-resolution transmission electron microscopy
Source: Strahlenther Onkol. 2020 Jan 31;196(9):821–33. doi: 10.1007/s00066-020-01576-1 (PMC7449954; doi:10.1007/s00066-020-01576-1)
Supplement: Supplementary file 5 — Table 5 PBLs of patients with head and neck cancer after RT and RCT were investigated using TEM before and 0.1, 0.5, and 24 h after the first fraction by quantifying pKu70 dimers and 53BP1 clusters in euchromatin and heterochromatin of 50 randomly chosen nuclear sections. Gold bead dimers and clusters were normalized to nuclear area and section thickness (pKu70 dimers/µm3 or 53BP1 clusters/µm3) and presented as mean values [file 66_2020_1576_MOESM5_ESM.pdf]

A	RCT patient [pKu70-dimers/µm^3]	RT patient [pKu70-dimers/µm^3]
**Time point after RT**		
	**Chromatin (total)**	
non-IR	0.11 ± 0.008	0.15 ± 0.014
0.1h	1.76 ± 0.035	1.75 ± 0.031
0.5h	2.09 ± 0.023	1.87 ± 0.025
24h	0.41 ± 0.014	0.24 ± 0.021
	**Euchromatin**	
non-IR	0.05 ± 0.007	0.06 ± 0.005
0.1h	1.53 ± 0.023	1.53 ± 0.030
0.5h	0.62 ± 0.016	0.49 ± 0.023
24h	0.18 ± 0.007	0.07 ± 0.012
	**Heterochromatin**	
non-IR	0.06 ±0.002	0.09 ± 0.013
0.1h	0.21 ± 0.024	0.22 ± 0.009
0.5h	1.47 ± 0.026	1.38 ± 0.011
24h	0.23 ± 0.013	0.23 ± 0.013

B	RCT patient [53BP1-clusters/µm^3]	RT patient [53BP1-clusters/µm^3]
**Time point after RT**		
	**Heterochromatin**	
non-IR	0.06 ± 0.004	0.09 ± 0.008
0.1h	0.21 ± 0.013	0.22 ± 0.011
0.5h	1.51 ± 0.046	1.43 ± 0.019
24h	0.50 ± 0.031	0.31 ± 0.013
